# NMR and proteomic analysis of *Cornus officinalis* extract reveals abundance of 5-hydroxymethylfurfural and induced expression of Nrf2 and superoxide dismutase-2 in 1.1B4 human pancreatic β-cells and murine islets

**DOI:** 10.1016/j.mce.2025.112675

**Published:** 2025-09-29

**Authors:** Justin D. Fletcher, Hailey L. Maurer, Mark A. Eschenfelder, Benjamin R. Smith, Sama Nayakanti, Jennifer Guergues, Tiara Wolf, Stanley M. Stevens, Brant R. Burkhardt

**Affiliations:** a Department of Molecular Biosciences, USA; b Department of Chemistry, University of South Florida, Tampa, FL 33620, USA

**Keywords:** Type 1 diabetes, *Cornus officinalis*, Non-obese diabetic mouse (NOD), 5-Hydroxymethylfuralfural, 1.1B4 cells, Nuclear magnetic resonance (NMR), High performance liquid chromatography (HPLC), Nrf2

## Abstract

We aimed to identify and isolate the metabolically active compounds from *Cornus officinalis* (*C. officinalis*) that underlie the biological effects previously observed in our in vitro and in vivo studies. Our prior findings demonstrated that *C. officinalis* promoted activation of the Keap1/Nrf2 pathway in a pancreatic β-cell line and delayed the onset of type 1 diabetes (T1D) in non-obese diabetic (NOD) mice. To characterize the metabolically active compounds within *C. officinalis*, the concentrated extract was fractionated by column chromatography, and evaluated for their effect on β-cell metabolic activity and expression of Nrf2 targets such as heme oxygenase-1 (HO1) and superoxide dismutase 2 (SOD2). Highly concentrated compounds of interest within metabolically active fractions were isolated using high performance liquid chromatography (HPLC). Elucidation of a highly abundant compound within *C. officinalis* extract was identified by mass spectrometry and nuclear magnetic resonance (NMR) as 5-hydroxymethylfurfural (5-HMF) which was demonstrated by immunoblotting to significantly increase SOD2 expression. Quantitative proteomics was performed on 5-HMF treated murine non-obese diabetic (NOD) islets and revealed increased expression of Nrf2 and downstream targets such as SOD2, GSTA3, GSTA4 and GCLC. Our findings suggest that 5-HMF is a highly abundant and metabolically active compound within *C. officinalis* that stimulates partial activation of the Keap1/Nrf2 pathway.

## 1. Introduction

Type 1 Diabetes (T1D) is an autoimmune disease initiated by a culmination of both genetic and environmental; these factors result in a cascading loss of pancreatic β-cells and insulin production, promoting dysglycemia, which is recognized as the symptomatic stage of T1D. Existing treatment focuses on insulin replacement and has been standard care for over 100 years with only one FDA approved T1D interventional therapy known as teplizumab, an anti-CD3 antibody that results in the exhaustion of CD8^+^ T cells (Herold et al., 2023). Teplizumab treatment was shown to have a significant effect on preservation of β-cell function two years after treatment but, unfortunately, the effect is greatly diminished after 7 years (Perdigoto et al., 2019). Investigation of new or supplemental interventional therapies is therefore needed to be used either alone or in conjunction with teplizumab.

One reason that teplizumab may not be completely successful as an interventional treatment for T1D may be due to the β-cell itself serving a critical role in T1D onset (Roep et al., 2021). The leading theory postulates β-cell stress precedes insulitis due to significant dysregulation of normal cellular process resulting in the creation of “neo antigens” (Piganelli et al., 2020). These neo antigens are created by several aberrant cellular processes in stressed β-cells and have been demonstrated to be immunogenic in T1D patients (Marre et al., 2018). With oxidative stress in the β-cell being directly linked to creation of neo antigens (Lichti and Wan, 2023), as well as β-cells having limited antioxidant capacity due to low antioxidant enzyme expression (Lenzen et al., 1996), compounds that can inhibit or help counteract oxidative stress in the β-cell may be beneficial in delaying or preventing T1D onset.

One such compound may be contained within the extract from the fruit of C. *officinalis*. Our prior findings have demonstrated that C. officinalis has beneficial effects on pancreatic β-cells both in vitro and in vivo. Specifically, we have shown that treatment with *C. officinalis* extract can promote cell viability, enhance oxidative capacity, and inhibit Th1 cytokine-mediated β-cell death in a pancreatic β-cell line (Sharp-Tawfik et al., 2019). Our extensive proteomic and molecular analysis identified *C. officinalis* induced phosphorylation of the selective autophagy receptor of p62 (Sequestosome-1/SQSTM1/p62) and activation of the antioxidant Kelch-like ECH-associated protein 1 (Keap1)/Nuclear factor-erythroid factor 2-related factor 2 (Nrf2) pathway. Several antioxidant proteins such as hemeoxygenase-1 (HO-1) and superoxide dismutase-2 (SOD-2) were found to be increased upon *C. officinalis* stimulation (Sharp-Tawfik et al., 2022). Furthermore, we demonstrated that treatment with *C. officinalis* extract was able to significantly delay T1D onset and pancreatic insulitis in the NOD mice (Fletcher et al., 2024). To elucidate the etiological agents within *C. officinalis* responsible for these beneficial effects, we sought to identify the specific compound inducing the biological effects observed within pancreatic β-cells.

To achieve this, we first evaluated the most abundant compounds within *C. officinalis* (loganin and morroniside) that have previously demonstrated biological activity by other investigators in non-pancreatic β-cell types (Cheng et al., 2020; Zhang et al., 2017, 2021; Kong et al., 2023; Wang et al., 2009; Li et al., 2023). After observing no significant biological effects from loganin or morroniside on our pancreatic 1.1B4 cell line, we then performed column chromatography separation using HP20 resin followed by high performance liquid chromatography (HPLC) fractionation to separate and purify the individual fractions of *C. officinalis*. This was then followed by examining the biological activity of the individual fractions via the MTT cellular viability assay. Both mass spectrometry and nuclear magnetic resonance (NMR) were performed to elucidate and identify our compound of interest as 5-hydroxymethylfurfural (5-HMF). We then performed immunoblotting and subsequent quantitative proteomic analysis to further validate and examine the impact of 5-HMF on Nrf2 driven antioxidant expression in pancreatic 1.1B4 cells and primary murine islets.

## 2. Materials and methods

### 2.1. Cell culture and treatments

The 1.1B4 cell line was originally commercially received (Sigma-Aldrich, Product Number 10012801–1VL) and cultured in RPMI media supplemented with 10 % Fetal Bovine serum and 1 % Penicillin streptomycin. Following seeding, cells were incubated overnight at 37 °C with 5 % CO_2_ to allow for cell adherence. After 24 h, cells were then applied treatment as described.

The NIT-1 cell line was commercially received (ATCC, Product Number CRL-2055) and cultured in F-12K medium supplemented with 10 % Fetal Bovine Serum and 1 % penicillin streptomycin. Following seeding, cells were incubated overnight at 37 °C with 5 % CO_2_ to allow for cell adherence. After 24 h, cells were then applied treatment.

### 2.2. Preparation of chemicals and reagents

*Cornus officinalis* (*C. officinalis*) was acquired by Evergreen Herbs (City of Industry, CA, USA). For cell culture treatment applications, 3 g of *C. officinalis* was dissolved in 12 ml of H_2_O and left on a rocker for 3 days to extract the compounds from *C. officinalis* into aqueous solution. The aqueous solution was then filtered using a 0.2 μm filter. The standards of Loganin (Cayman chemicals, Item No. 19997) and Morroniside (Cayman chemicals, Item No. 30711) were prepared by resuspending the solutions in water then filter sterilizing using a 0.2 μm filter. All other chemicals were of analytical grade and commercially available. 5-(Hydroxymethyl)furfural was purchased from Sigma Aldrich (Cat No. W501808) and resuspended in H_2_O at a concentration of 5 mg/ml.

### 2.3. Column chromatography

To generate the *C. officinalis* extraction for column chromatography analysis, 100 g of *C. officinalis* was put on a rocker with 400 mL of H_2_O overnight. The supernatant was then collected with this procedure performed 2 more times. Following the 3 extractions, the individual collected supernatants were combined and then loaded onto a chromatography column containing 200 g of Diaion™ HP20 (ThermoFischer, ItemNo. 046488.A1). The wash through of the column was then discarded, and gradient elution was then performed. Each elution consisted of 400 mL of a methanol solution with the concentration increasing by 10 % with each fraction collected. 400 mL fractions were then collected for each 10 % fraction. After collection fractions were left to evaporate overnight to remove the organic solvent and then poured into cube molds and put into a −80 °C freezer. After the samples were frozen, they were lyophilized, and the resulting powder was then resuspended in 20 mL of water to use for further analysis and purification.

### 2.4. Analytical HPLC analysis

HPLC analyses were performed using a Hitachi LaChrom Ultra HPLC system (Hitachi Ltd, Chiyoda City, Tokyo, Japan) with a diode array detector. The monitoring wavelength was set at 240 nm. A Hitachi LaChrom C18 column (150 mm × 4.6 mm, 5 μm) was used with a flow rate of 1.0 mL/min. The mobile phase consisted of water (Solvent A), and Methanol (Solvent B) starting with an EQ at 5 % for 1 min then using a gradient elution 5–70 % solvent B from 1 to 15 min followed by a wash with 100 % methanol for 1 min. The column temperature was maintained at 30 °C, and 10 μL aliquots of the supernatants were injected into the LaChrom C18 column.

### 2.5. ELISA analysis of HO1

Cells were seeded in a 12 well plate in 1 ml of a 1 × 10^5^ cells/ml solution. Cells were then allowed to attach overnight and treated with 250 μl of the respective *C. officinalis* fractions, Whole *C. officinalis* extract (CO), water (VC) and 100 μm of loganin and morroniside with remaining untreated cells (NT) used as a control. After 4 h the media was removed from the wells and cells were washed with PBS and lysed using M-PER lysis buffer (Thermo Fischer Scientific, Item # 78501). An HO1 ELISA was then performed using a commercially available kit and procedures were followed according to the manufacturer’s instructions (Thermo Fischer Scientific, Cat. # EEL060).

### 2.6. MTT assay

Cells were seeded in a 96 well plate with 100 μl of a 5 × 10^4^ cell/mL suspension in each well. Cells were then left untreated (NT group), treated with water (Vehicle control (VC) and then the respective treatments. After the initial treatments, the MTT assay (Promega, Item No. G4000) was performed. In brief, the MTT dye (3-(4,5-Dimethylthiazol-2-yl)-2,5-Diphenyltetrazolium Bromide) was added in a 15:100 ratio for each well and incubated at 30 °C for 4 h. The solubilization solution was then added in a 1:1 ratio and incubated overnight. Absorbance of each well of the 96 well plate was then measured at 570 nm. Statistical analysis was performed by one-way analysis of variance (ANOVA) comparing the treated groups to the NT group followed by Dunnett’s multiple comparisons test using GraphPad Prism version 10.0.3 for Windows, GraphPad Software, Boston, Massachusetts USA, www.graphpad.com.

### 2.7. HPLC purification of the RT 4.09 peak isolated from C. officinalis extract

HPLC purification of the RT 4.09 peak was performed using an Agilent Technologies 1260 Infinity HPLC system (Aligent Technologies Inc., Santa Clara, California) with the monitoring wavelength set at 240 nm. Crude extract of *C. officinalis* was fractionated by C18 HPLC (Gemini C18, 110 Å, 250 mm × 21.2 mm) using a 26 min gradient of 5–100 % H_2_O/CH_3_OH followed by 100 % CH_3_OH for 4 min. Separation was held at 12 mL/min at 30 °C and UV detection at 240 with fractions collected from HPLC, lyophilized, and resuspended in nanopure water for use in cell culture treatments.

### 2.8. Mass spectrometry

All samples were analyzed via single-quadrupole mass spectrometer coupled with liquid chromatography. HPLC-grade H_2_O and CH_3_CN were used as solvents A and B, respectively. Both solvents contained 0.1 % formic acid to ensure the analytes were fully protonated. A gradient was used starting at 10 % solvent B to 100 % B over 8 min with a flow rate of 1.3 mL/min. The diode array detector was set to measure wavelengths 254, 230, and 280 nm. Samples were first filtered through a 0.45 μm filter, and 1 μL was injected into a Phenomenex Poroshell 120 EC-C18 2.7 μm column with dimensions 4.6 × 30mm. Temperature was kept constant at 30 °C. The mass spectrometer detector was set to API-ES ionization mode with both positive and negative polarities.

### 2.9. Nuclear magnetic resonance spectroscopy

1H and 13C NMR spectra were obtained on a Varian 600 MHz broadband instrument operating at 600 MHz for 1H and 150 MHz for 13C. After lyophilization, the samples were fully dissolved in deuterated methanol (Sigma-Aldrich) at a concentration of 3 mg/ml before adding to a standard 3 mm NMR tube. The residual solvent peaks were used as internal references (δH 3.31 for 1H and δC 49.1 for 13C).

### 2.10. Western blotting

Cells were seeded on a 6 well plate and treated with *C. officinalis* extract. Cells were pelleted and resuspended in lysis buffer (50 mM ammonium bicarbonate solution with 50 mg of SDS). Lysates were heated at 95 °C for 4 min and sonicated for 6 rounds at 200 amp for 3 s each round. Protein concentrations were quantified using the Pierce 660 nm protein assay (Thermo Fisher Scientific). Protein samples were electrophoresed on a 4–20 % TGX SDS PAGE gel (Bio-Rad) using the Mini-PROTEAN Tetra Vertical Electrophoresis Cell (Bio-Rad) and transferred to a nitrocellulose membrane using the iBlot dry transfer system (Invitrogen). The nitrocellulose membrane was blocked using 5 % BSA-Tris-buffered saline with 0.1 % Tween® 20 Detergent (TBST) for 1 h. The membranes were incubated overnight with primary antibodies for the detection of SOD2 (Cell Signaling Technology #13141T, 1:500) and Beta Actin (Cell Signaling Technology #4907S). Membranes were then incubated in HRP conjugated secondary antibody (Cell Signaling Technologies #7074P2) followed by chemiluminescent detection (SuperSignal West Femto, Thermo Fisher Scientific). Membranes were then imaged using the Amersham™ Imager 600 (GE Healthcare Life Sciences). Densitometric analysis for measurement of pixel density from band detection upon western analysis was performed using ImageJ version 1.53r (Schneider et al., 2012).

### 2.11. Animal husbandry

NOD/ShiLtJ (Strain #: 001976) 6-week-old female mice were purchased from Jackson Laboratories (Bar Harbor, ME, USA) and housed at 25 °C and 60 % humidity and maintained on a 12-h light/dark cycle in the USF College of Medicine vivarium. Mice had unlimited access to laboratory chow (Research diets, 24 % protein, 41 % carbohydrates, and 24 % fat) Husbandry and experimental procedures were reviewed and approved by the USF Institutional Animal Care and Use Committee under Protocol #IS00013350.

### 2.12. Isolation of primary murine islets

Pancreatic islets were isolated from female NOD mice between 6 and 8 weeks old. Animals were obtained from Jackson Laboratories and housed in the USF College of Medicine Vivarium prior to islet isolation. Husbandry and experimental procedures were reviewed and approved by the USF Institutional Animal Care and Use Committee under Protocol #IS00013350. Immediately preceding islet isolations, non-fasting blood glucose levels were assessed using blood obtained through a tail prick and measured using a Contour Next glucometer; healthy, non-diabetic mice were verified as those with non-fasting glucose levels less than 250 mg/dL. Mice were euthanized by exposure to CO_2_ prior to pancreas extraction.

Islet isolation was performed using a modified collagenase digestion method (Villarreal et al., 2019). Collagenase P solution (Sigma-Aldrich) containing collagenase P enzyme and bovine serum albumin was carefully injected into the endocrine pancreas through the bile duct, inflating the pancreas. The inflated pancreas was extracted and placed in additional collagenase P for a digestion period of 11.5 min.

The digested pancreas was washed thoroughly with STOP solution containing fetal bovine serum and additional washes were done using Hanks’ Balanced Salt solution. Islets were separated from the digested tissue mixture using a double filtration system to remove large undigested tissue with a 400 μm nylon filter (PluriSelect Life Science) and small non-islet cells with a 100 μm nylon filter (Bio Basic) (Salvalaggio et al., 2002).

Islets were incubated at 37 °C, 5 % CO_2_ in RPMI 1640 medium containing 10 % fetal bovine serum, 100 U/mL penicillin, 100 μg/mL streptomycin, 5.5 mM glucose, and INS-1 cell supplement. After a 12-h recovery period, viable islets with a rounded, golden appearance were selected under a dissection scope (Meiji) and added to a non-treated dish containing media. We found that murine islets can be maintained in culture for up to 4–5 days following isolation as previously documented (Corbin et al., 2021).

### 2.13. Preparation of 5-HMF treated islet samples for proteomic analysis

1200 islets were isolated as described. Following a 12-h recovery period, islets were transferred to non-treated 6-well plates. 12 wells were used in total, each containing approximately 100 islets in 1 mL RPMI 1640 islet medium. Treatments included a no treatment group, a high-dose 5HMF-treated group (1600 μg/mL), and a low-dose 5HMF-treated group (800 μg/mL); each treatment group included four replicates. Islets were incubated with treatments at 37 °C, 5 % CO2 for 18 h.

### 2.14. Proteomic analysis of 5HMF-treated islet samples

Frozen pancreatic islet pellets (n = 4 per group) were processed for mass spectrometry using the iST kit (PreOmics GmbH, Planegg/Martinsried, Germany) as previously described (Gebru et al., 2024). Briefly, 50 μL of kit-provided lysis buffer was added to each sample followed by probe sonication until samples were no longer viscous (20 % amplitude, 6 cycles, 5 s on/off). The resulting protein was assessed using the Pierce 660 nm Protein Assay (ThermoFisher, Waltham, MA) on a microplate reader. The protein amount and volume were normalized across all samples prior to digestion and peptide purification as described by kit instruction, aside from a modification where samples are digested prior to cartridge loading (Kulak et al., 2014). The final eluates were dried down in a vacuum concentrator prior to resuspension in 0.1 % formic acid in LC-MS-grade H_2_O. Peptide concentrations were measured on a NanoDrop spectrophotometer (ThermoFisher) prior to analysis of the digested pancreatic islet lysates using a timsTOF Pro mass spectrometer coupled in-line to a NanoElute 2 UHPLC system (Bruker, Billerica, MA). Ionization was performed using a CaptiveSpray ion source, with the attached column oven maintained at 50 °C and containing an Aurora Ultimate CSI UHPLC reversed-phase C18 column (25 cm × 75 μm i.d., 1.7 μm C18, IonOpticks, Fitzroy, Australia). Mobile phase A (0.1 % formic acid in water) and mobile phase B (0.1 % formic acid in acetonitrile) were applied in a 60-min gradient from 2 % to 25 % B. The method included an additional ramp to 25–37-90 % B followed by 10 min of 90 % B to wash the column between injections, totaling an 80-min run time. Data were acquired in diaPASEF mode, covering an ion mobility range of 0.70–1.40 1/K_0_ [V⋅s/cm^2^] with a cycle time of ~1.48 s. Prior to acquisition, linear calibration of both ion mobility and m/z were performed using three ions at 622, 922, and 1222 m/z (Agilent, Santa Clara, CA).

Data were searched with DIA-NN (v. 2.2.0) using a predicted library generated from the UniProt Mus Musculus database (63208 entries: When downloaded?). Label-free quantification (LFQ) was utilized with match-between-runs (MBR) applied, using a 1 % protein and precursor FDR cutoff with additional run-specific 1 % FDR filter at the protein level (using –matrix-spec-q 0.01 command). Furthermore, reannotate, mass and MS1 accuracy both set at 15, generic scoring, genes proteo-typicity, NNs (cross-validated) machine learning, quantUMS (high precision), RT-dependent cross-run normalization, and IDs RT &IM profiling were selected.

Differential expression analysis was conducted in MS-DAP (v. 1.2.2) (Koopmans et al., 2023)within RStudio where the DIA-NN output and library files were input with appropriate sample metadata. Contrasts were setup to include notx (no treatment control) to 5hmf_lo (800 mg/ml 5-HMF: Brant’s lab to add), notx to 5hmf_hi (1600 mg/ml 5-HMF: Brant’s lab to add), and 5hm5_lo to 5hmf_hi for differential expression analysis using the algorithm MS-EmpiRe (Ammar et al., 2019) Minimum detection and quantification filters of at least 3 out of 4 replicates were applied with additional filter fraction detection and quantification filter set for at least 75 % of samples per group. Variance Stabilizing Normalization (VSN) and Mode Between protein (MBprot) normalization steps were applied sequentially prior to differential expression analysis. The MS-EmpiRe q-value threshold was set at 0.01 and log2 foldchange threshold was set to automatically infer through bootstrapping, which is the filtering basis to achieve a statistically robust list of differentially expressed proteins (DEPs labeled as “TRUE” in output).

### 2.15. ELISA analysis of secreted insulin

NIT-1 cells were seeded as described in section 2.1. Treatments included a no treatment group, a vehicle control group with nanopure water, a high-dose 5HMF-treated group with concentration of 1600 μg/mL, and a low-dose *C. officinalis*-treated group with concentration of 800 μg/mL. After a 4-h treatment, media was collected and insulin concentration was measured with a commercial insulin ELISA assay following procedures according to manufacturer’s instructions (Crystal Chem, Cat. # 90080).

## 3. Results

### 3.1. Morroniside and loganin did not increase 1.1B4 pancreatic β-cell viability

**Fig. 1.**
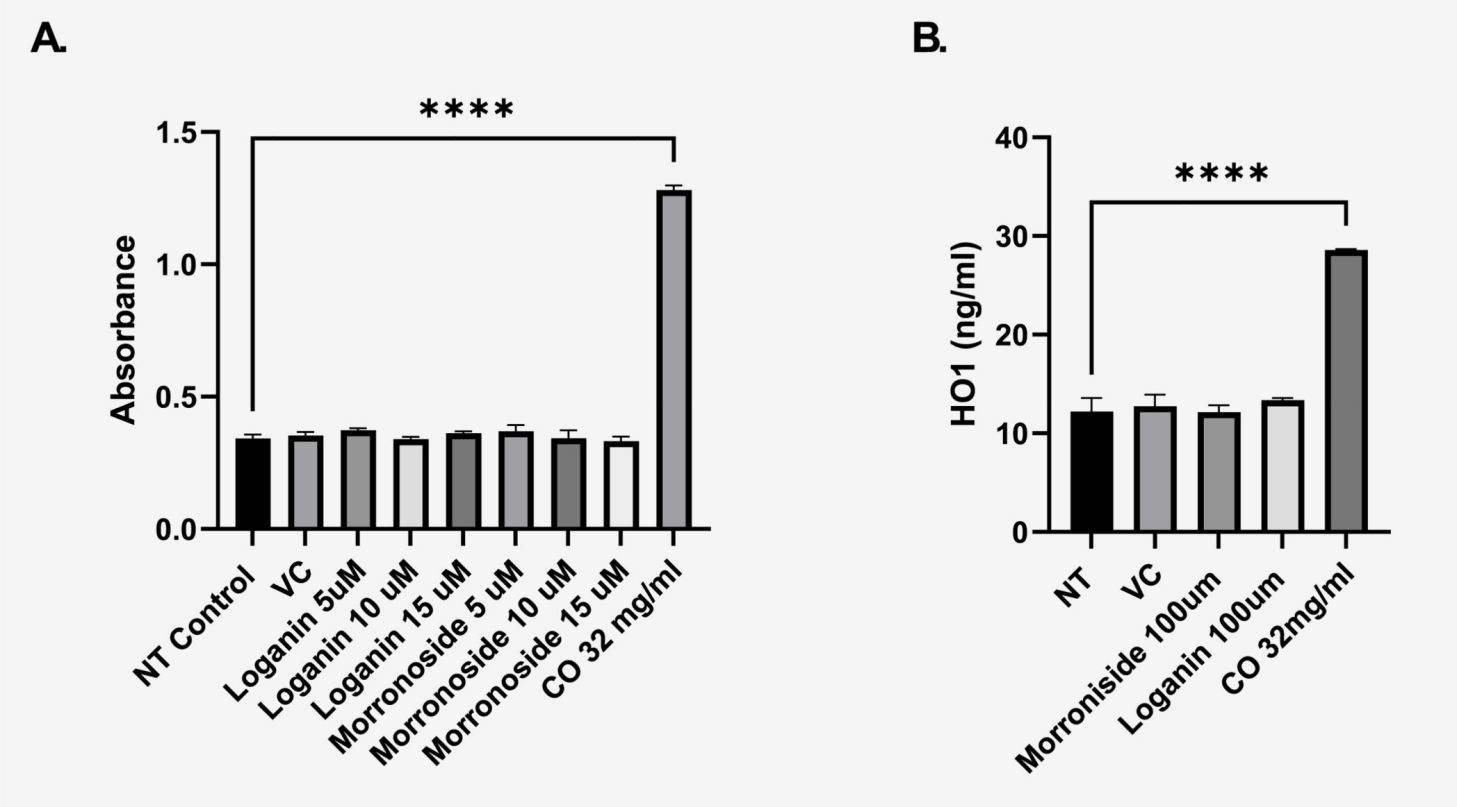
Loganin and morroniside do not increase viability or HO-1 expression in the pancreatic 1.1B4 cells. **(A)** MTT assay with the absorbance measured at 570 nm after the designated treatments on the X axis. Data are shown as mean ± SEM. **** denotes statistical significance (P < 0.0001) as determined by one way ANOVA test. Post hoc analysis performed by Dunnett’s multiple comparisons test. **(B)** Whole cell lysate was collected after corresponding treatments and HO1 concentration was determined by commercial ELISA. Average HO1 levels are shown as mean ± SEM. P < 0.05 as determined by one way ANOVA test (N = 3) Post hoc analysis performed by Dunnett’s multiple comparisons test.

Our prior published results detailed the biological effects of *C. officinalis* extract both in vivo and in vitro (Sharp-Tawfik et al., 2019, 2022; Fletcher et al., 2024) but the precise compounds inducing this biological effect in the 1.1B4 pancreatic β-cell line were unknown. Prior literature from our laboratory and others indicated significant abundance of both loganin and morroniside within the *C. officinalis* extract (Gao et al., 2021). In terms of biological activity, morroniside has been shown to activate the Keap1/Nrf2 pathway in the mouse brain (Li et al., 2023) and decrease AGE-RAGE signaling in a T1D mouse model (Sun et al., 2020); loganin can promote a beneficial hypoglycemic effect through the promotion of glucose uptake in a streptozotocin (STZ) induced diabetic mouse model (He et al., 2016). Since loganin and morroniside appear to be the most abundant and well-studied individual constituents within *C. officinalis*, we therefore examined these top 2 candidates in the context of our study. We performed an MTT assay evaluating loganin and morroniside stimulated 1.1B4 cells and did not observe any significant changes as compared to that of *C. officinalis* (Fig. 1A). Furthermore, as previously discussed, morroniside has been shown to induce the Keap1/Nrf2 pathway in vivo (Li et al., 2023), To further investigate if morroniside was responsible for *C. officinalis’s* biological effect inducing Keap1/Nrf2, we assessed its potential to activate this pathway in the 1.1B4 cell line. This was assessed by measuring a predominant downstream target of the Keap1/Nrf2 pathway by ELISA, Heme oxygenase-1 (HO1) (Guerrero-Hue et al., 2020). Surprisingly, morroniside had no significant effect on HO1 expression in the 1.1B4 cells. However, whole extract of *C. officinalis* (CO) (Fig. 1B) significantly increased HO-1 expression as determined by ELISA, indicating the biological effect of *C. officinalis* on the 1.1B4 cells does not appear to be caused by either loganin or morroniside.

### 3.2. Analytical HPLC analysis of C. officinalis extract

**Fig. 2.**
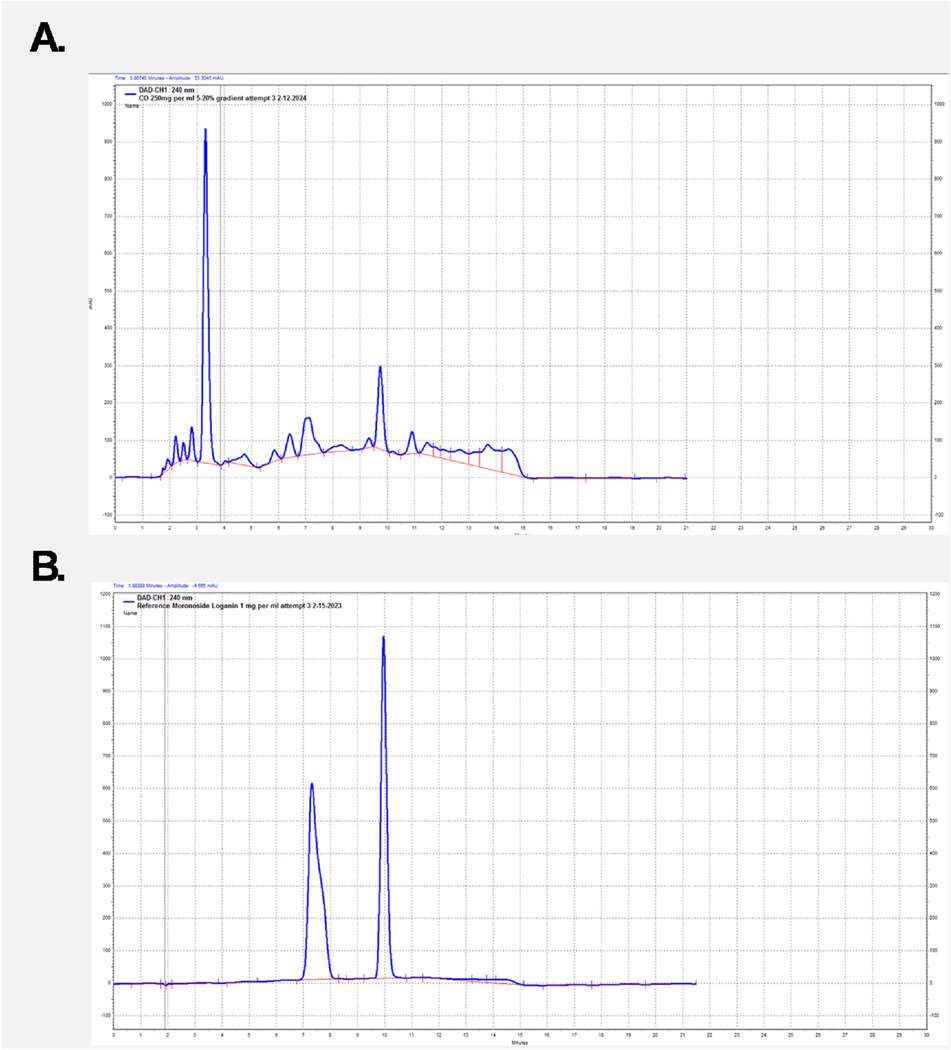
Analytical HPLC chromatograms of CO and Loganin and Morroniside standards **(A)** HPLC chromatogram of whole CO extract (250 mg/ml). Absorance measured at 240 nm **(B)** HPLC chromatogram of the morroniside and loganin standards (1 mg/ml). Absorbance measured at 240 nm.

Since morroniside or loganin did not promote cell viability, we explored the identity of other compounds within the *C. officinalis* extraction responsible for the induced biological activity. Analytical HPLC of the *C. officinalis* extraction was performed and peak intensities were measured. In the complete *C. officinalis* extraction, both morroniside and loganin were found to have discrete independent peaks with the loganin peak having higher intensity than morroniside (Fig. 2A). As shown in the HPLC chromatograms as compared with purified loganin and morroniside standards, morroniside and loganin standards elute at a retention time (RT) of 7.3 and 9.8 min, respectively (Fig. 2B). As anticipated, loganin and morroniside are among the most abundant compounds but there was an additional prominent peak identified at a RT of 3.3 (Fig. 2A). We then further extrapolated the abundant compounds within this peak to identify and determine if these individual constituents are responsible for the observed biological activity of *C. officinalis*.

### 3.3. Separation of individual compounds of C. officinalis using column chromatography and HPLC reveal new biologically active compounds

**Fig. 3.**
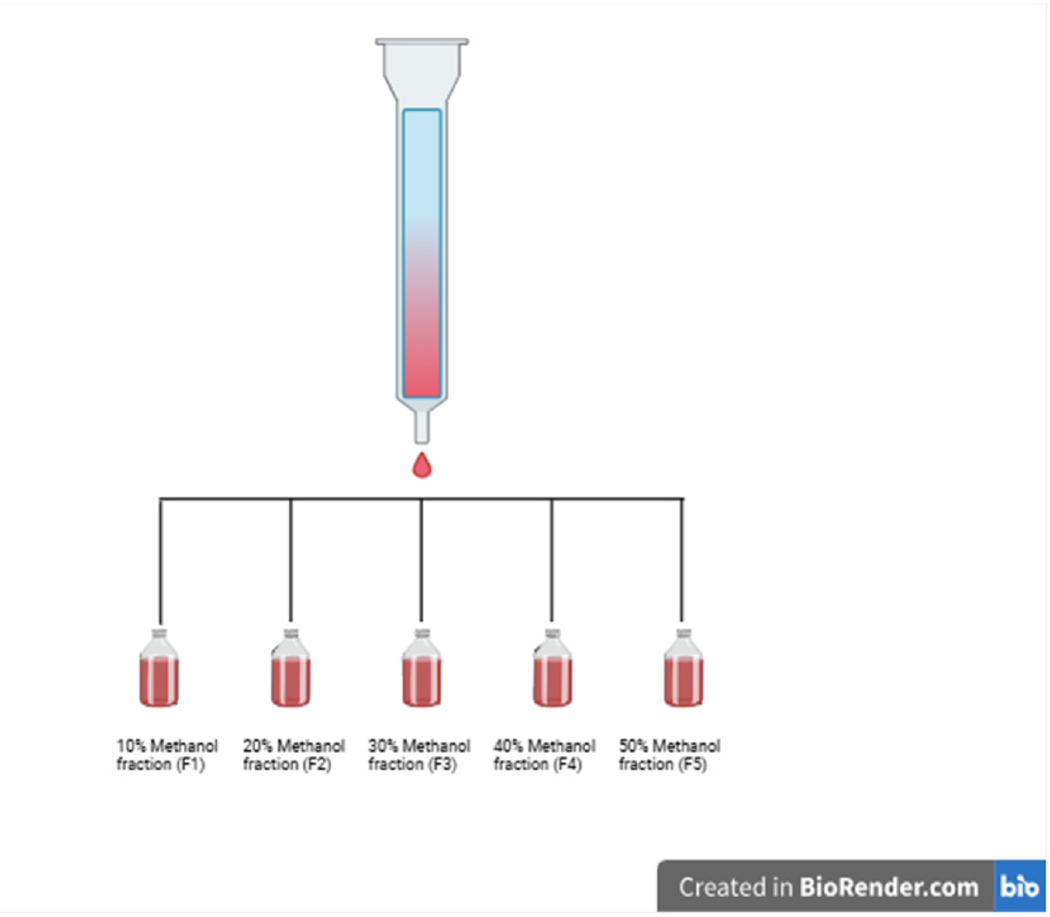
Schematic representing column chromatography fractionation of the C. officinalis extraction. As the methanol concentration increased by 10 % a fraction was collected corresponding with the F1-F5 fractions. Image was created in biorender. Created in BioRender. Fletcher, J. (2025) https://BioRender.com/l32w974.

**Fig. 4.**
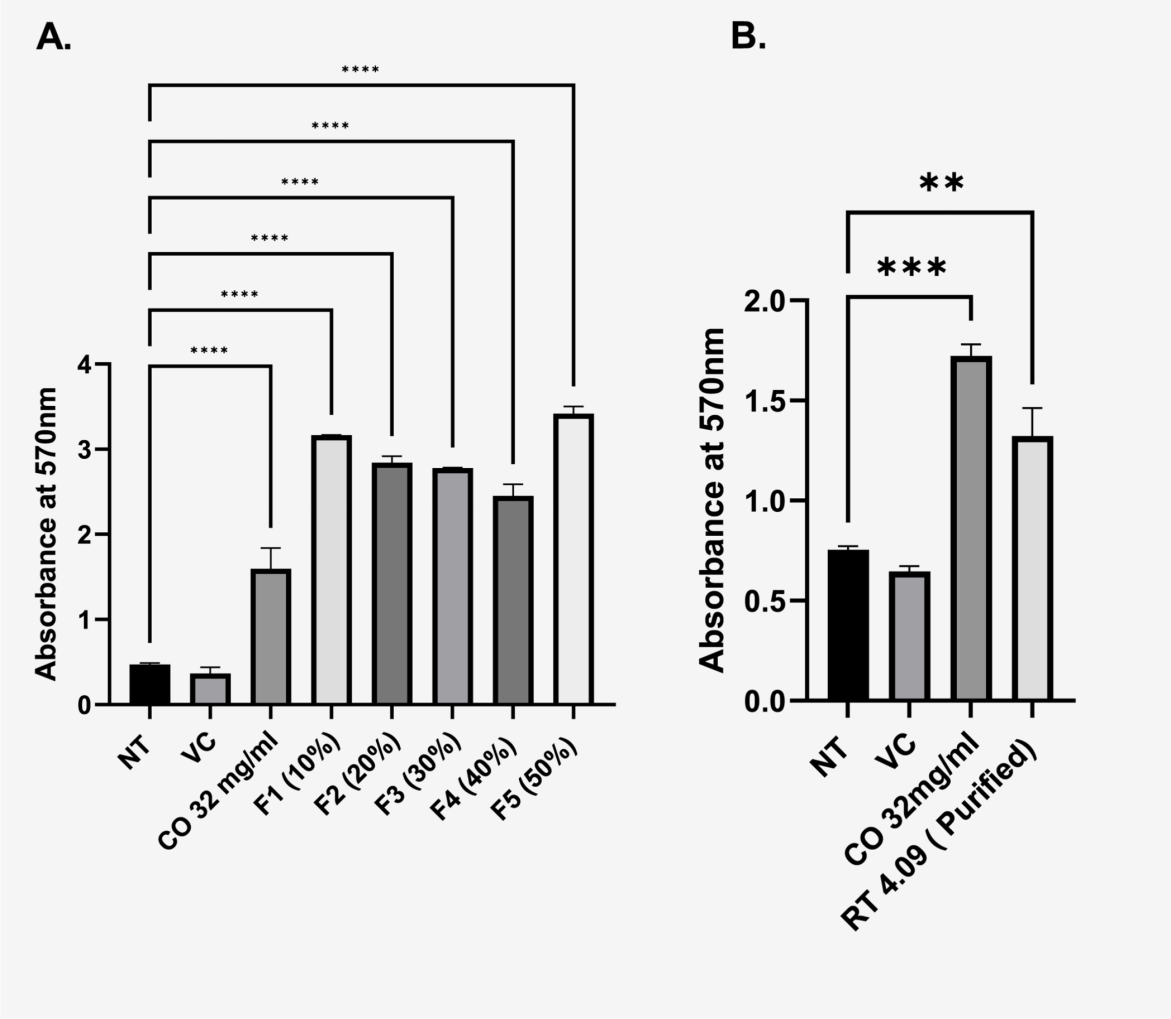
Individual Fractions isolated from *C. officinalis* (CO) have a dose dependent effect on viability as measured by MTT on the 1.1B4 cells. **(A)** MTT assay for the unpurified fractions with the absorbance measured at 570 nm after the designated treatments on the X axis. **(B)** MTT assay for the RT 4.09 fraction after purification by HPLC with the absorbance measured at 570 nm after the designated treatments on the X axis. Data are shown as mean ± SEM. **** denotes statistical significance (P < 0.0001) as determined by one way ANOVA test Post hoc analysis performed by Dunnett’s multiple comparisons test (N = 2). NT-No treatment applied and VC-Vehicle control (Water) control.

**Fig. 5.**
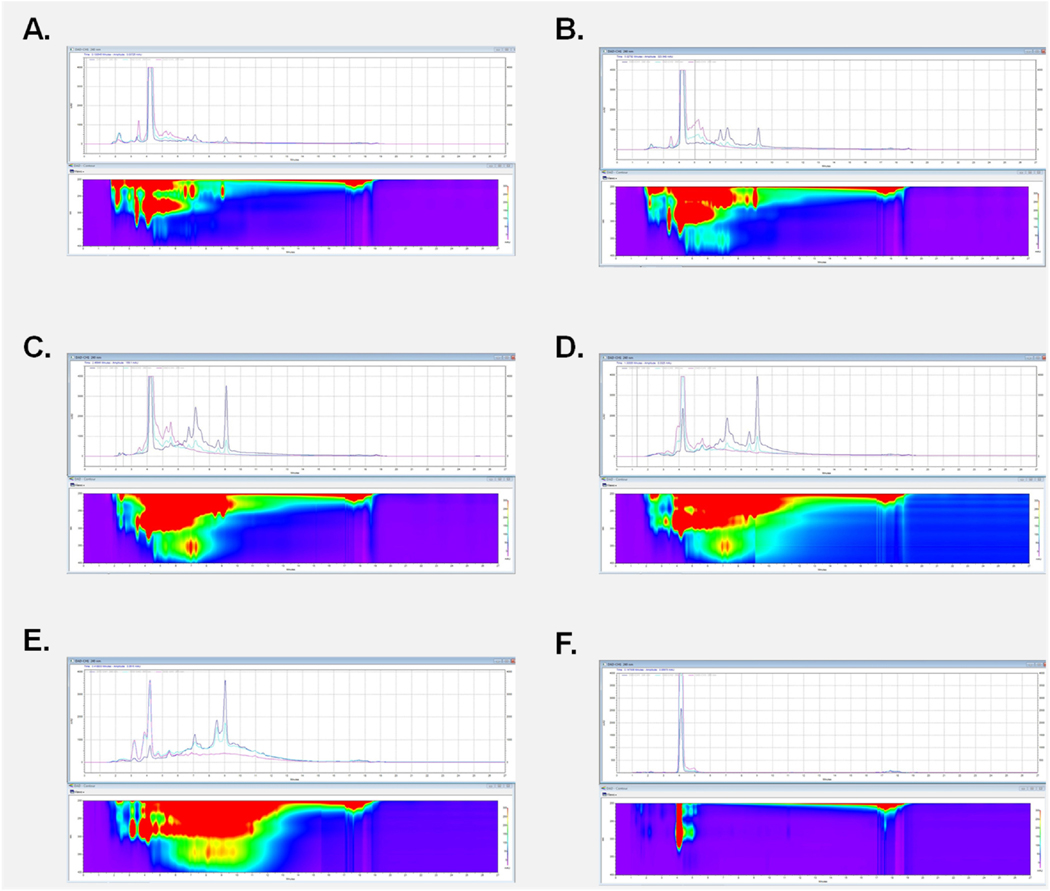
HPLC and Diode array Detector analysis of individual *C. officinalis* (CO) fractions. Wavelengths on DAD range from 200 to 400 nm. HPLC chromatogram and DAD heat maps of the following F1 to F5 fractions: **(A)** F1, **(B)** F2, **(C)** F3, **(D)** F4, **(E)** F5 fraction. **(F)** HPLC chromatogram and DAD heat map of F1 after purification using a preparative HPLC.

To start our fractionation, we used a column chromatography approach using an HP 20 solid phase to separate our polar from non-polar compounds. After the column chromatography, 5 fractions were collected (F1-F5) at proportionally increasing concentrations of methanol (Fig. 3). Following the column chromatography, we determined which fractions contain the biologically active constituents responsible for *C. officinalis*’s biological effect. Our prior findings demonstrated that *C. officinalis* increased cellular viability as measured by MTT (Sharp-Tawfik et al., 2019), For this reason we tested each individual fraction by MTT. Collected fractions were lyophilized by vacuum drying and then resuspended in water to then be applied to the 1.1B4 pancreatic cells followed by measurement of MTT. Application of fractions F1 through F5 significantly increased viability (Fig. 4). Interestingly, fractions F2 through F4 all had a progressively diminished, yet significant effect, on viability indicating a potential dose response (Fig. 4). Analytical HPLC coupled to a diode array detector was performed to identify peaks with identical retention times (RT) in the F1-F5 fractions (Fig. 5A–E). All 5 fractions contained a prominent peak with a retention time of 4.09 min that decreased in intensity until Fraction 5 where it was no longer the most abundant compound. Considering the RT 4.09 peak is the most abundant in F1-F4 and follows the dose response shown in Fig. 4 we attempted to purify this peak further and evaluate for biological effect. Although the column chromatography had separated out many of the polar from non-polar compounds in *C. officinalis* the DAD heatmaps of our fractions (Fig. 5A–E) clearly showed that further purification was required. To purify the RT 4.09 peak further, we decided to use a preparative HPLC approach with a C18 column to isolate the RT 4.09 peak. After the HPLC fractionation, we further isolated the RT 4.09 peak by HPLC (Fig. 5F) resulting in a purified fraction with a percent area of 97.37 % (Fig. 12) indicating a relatively high level of purity. This fraction was then used for biological testing and structural determination. To test the biological activity of this fraction, we performed the MTT assay utilizing the purified RT 4.09 sample and revealed an increase in viability (Fig. 4B) in 1.1B4 cells as compared to the whole *C. officinalis* extract and the unpurified fraction (Fig. 4A) indicating that the RT 4.09 peak may contain the compound responsible for *C. officinalis*’s biological effect as observed in the 1.1B4 cells.

### 3.4. Structural determination of the RT 4.09 peak reveals 5-hydroxymethylfurfural (5-HMF)

**Fig. 6.**
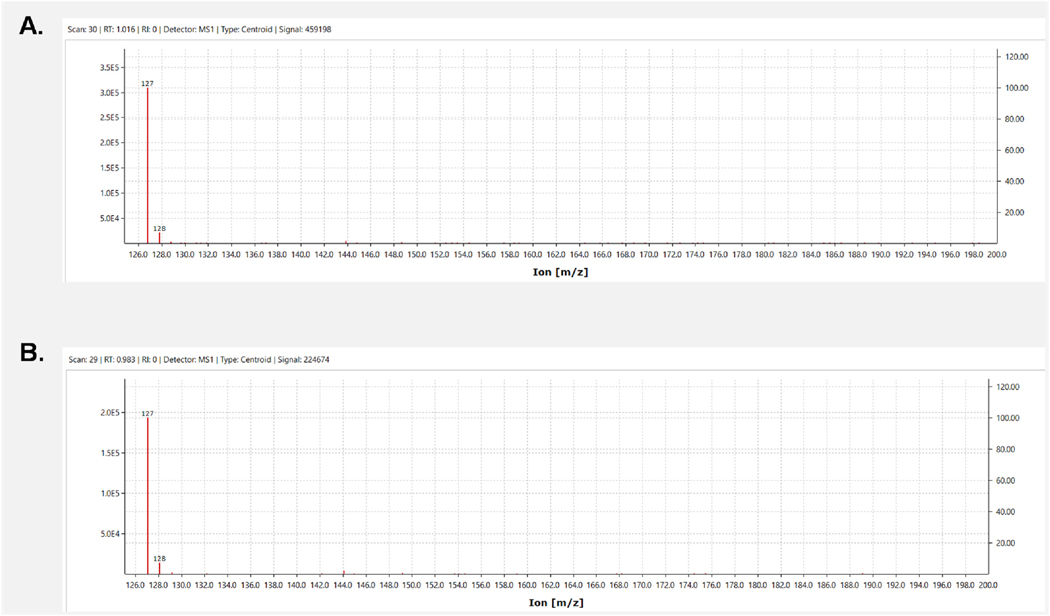
Mass spectrum of 5-HMF and the RT 4.09 sample.

To identify the compound present in the RT 4.09 sample, we utilized an MS approach to determine the molecular weight of the unknown compound. Initial MS analysis revealed the most prominent peak in the MS spectra at M/z 127 (Fig. 6A). As the chemical composition of *C. officinalis* has been extensively published (Yokozawa et al., 2010; Dong et al., 2018), we examined the available compositional database of *C. officinalis* to ascertain which compound had a protonated adduct [M + H] of 127. Our investigation revealed a furan in *C. officinalis* called 5-hydroxymethylfurfural (5-HMF) that had a mass of 126.03 (Dong et al., 2018). As this was very similar to our mass, we obtained a commercially available 5-HMF and performed a single quadrupole mass spectrometer analysis. We found that the mass spectra for 5-HMF (Fig. 6B) was virtually identical to our RT 4.09 sample.

**Fig. 7.**
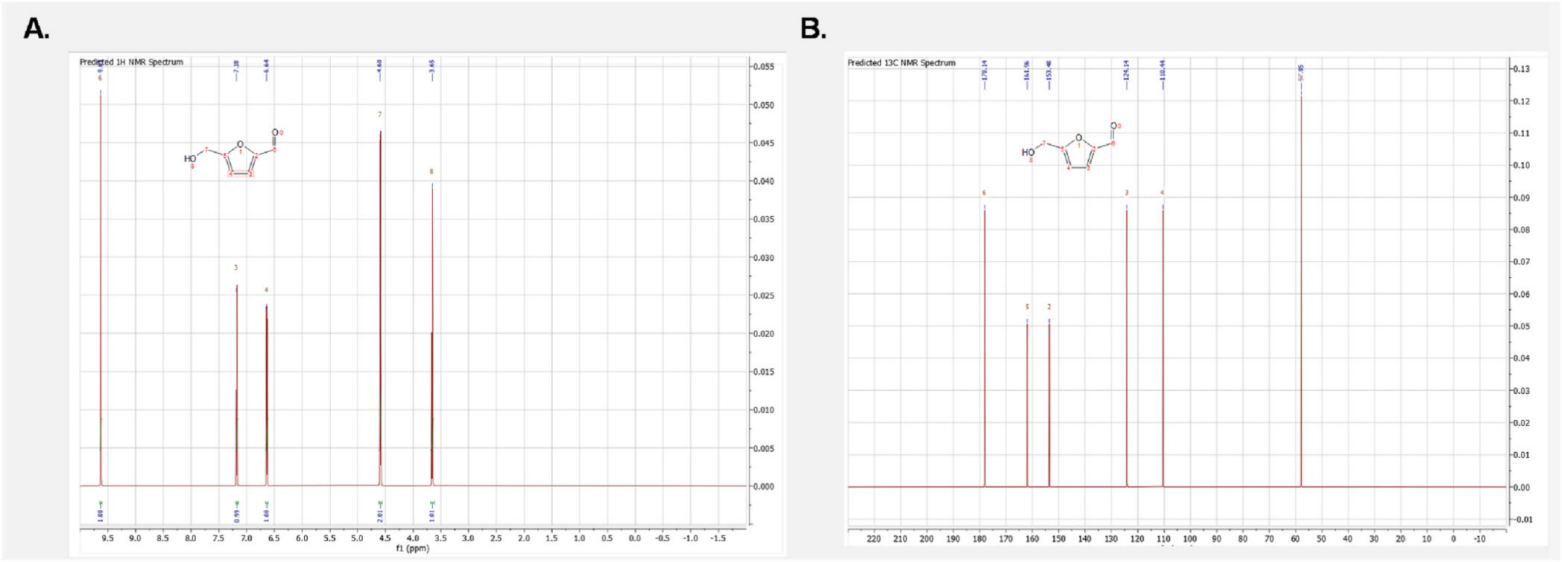
Predicted Structure and chemical shifts for 5 HMF in both HNMR and CNMR **(A)** Predicted HNMR chemical shift for 5HMF with the molecular structure numerically labeled to the corresponding HNMR peaks **(B)** Predicted CNMR chemical shift for 5HMF with the molecular structure numerically labeled to the corresponding CNMR peaks.

**Fig. 8.**
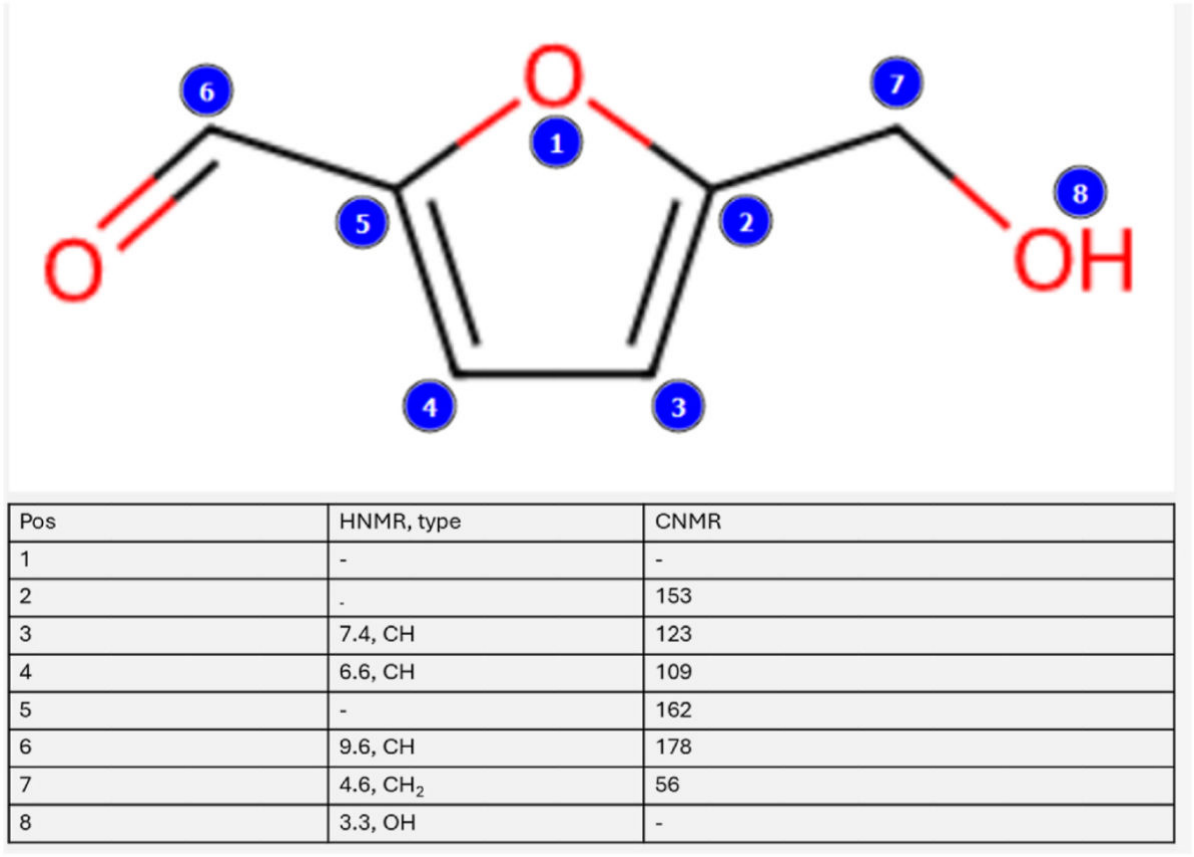
Predicted chemical shift values for 5-HMF drawing of the molecular structure of 5-HMF with the corresponding chemical shift values for the labeled locations (In blue) listed in the table.

**Fig. 9.**
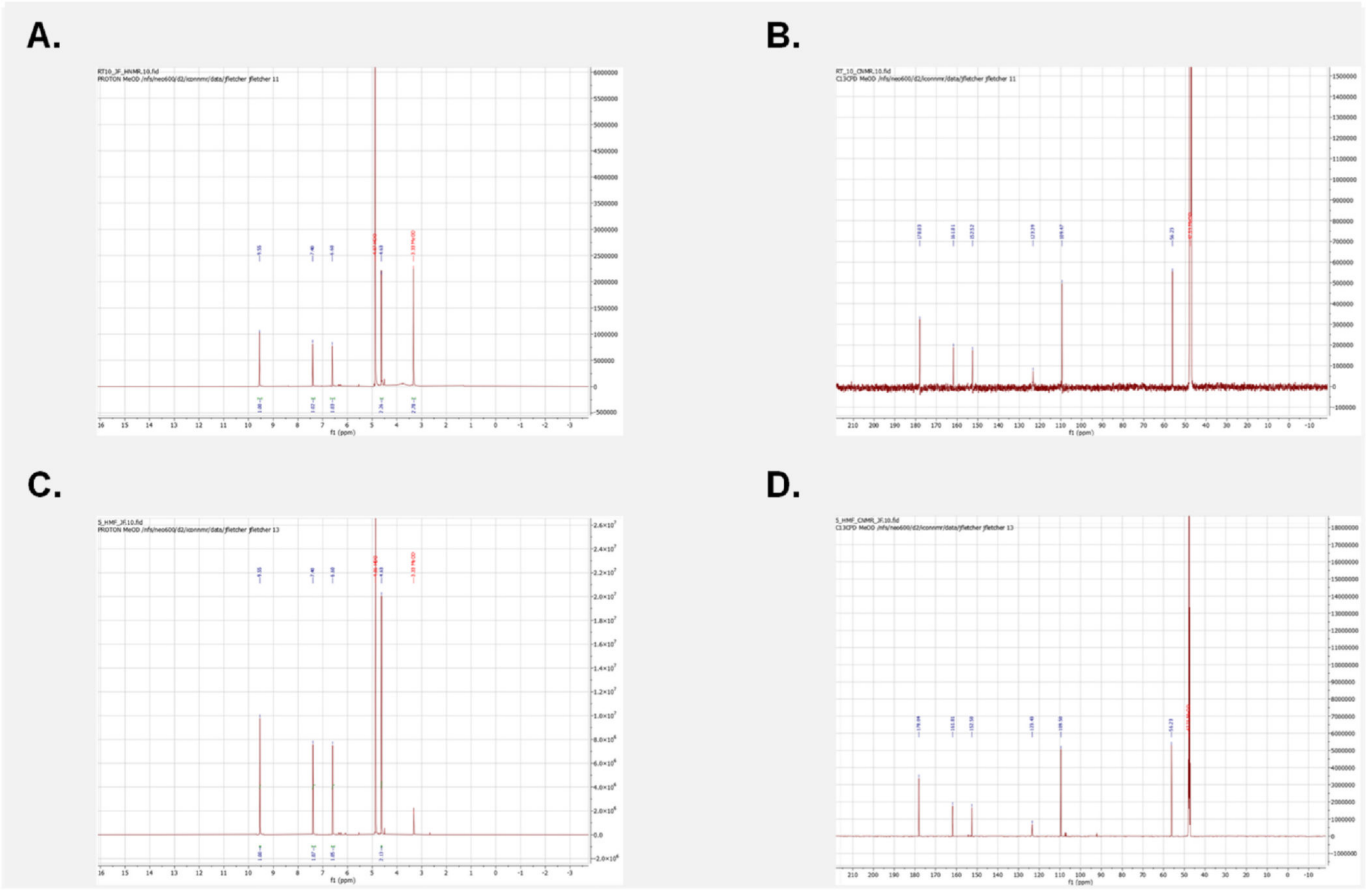
HNMR and CNMR spectra for both 5-HMF and the RT 4.09 sample. **(A)** HNMR spectra for the RT 4.09 sample **(B)** CNMR spectra for the RT 4.09 sample **(C)** HNMR spectra for the purchased 5-HMF **(D)** CNMR spectra of commercially received 5-HMF.

**Fig. 10.**
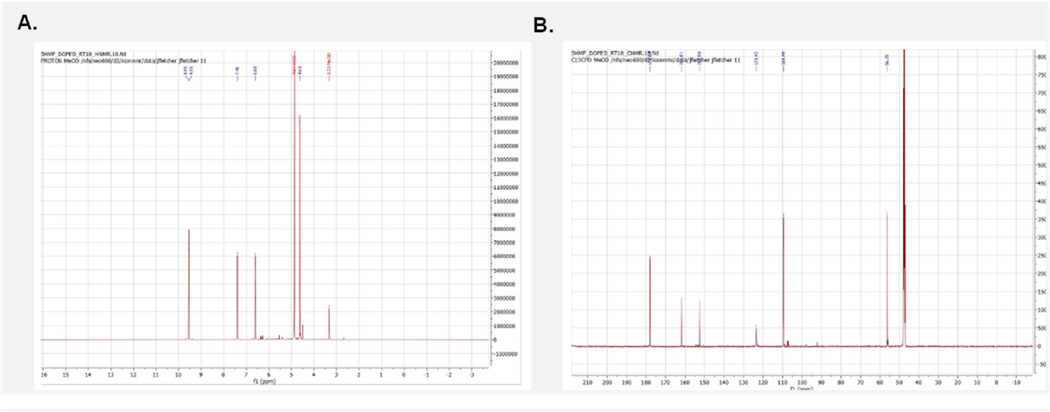
HNMR and CNMR spectra for the 5-HMF sample spiked with RT 4.09 sample. **(A)** HNMR spectra for the spiked 5-HMF sample (**B)** CNMR spectra for the spiked 5-HMF sample.

To confirm the structure of our RT 4.09 sample as 5-HMF we further validated utilizing an NMR approach. This was initiated by employing the MestRenova (version 15.1, Mestlab Research) prediction software to obtain the general chemical shifts for both the carbons and hydrogens present in 5-HMF (Fig. 7) with the numerical values listed for both the predicted CNMR and HNMR (Fig. 8). Of particular note is the prominent aldehyde peak with an HNMR chemical shift of 9.6 and a CNMR chemical shift of 178 as this is very unique to 5-HMF when compared to the other compounds found in *C. officinalis* (Dong et al., 2018). Following the determination of the general expected NMR peaks for 5-HMF, we performed both CNMR and HNMR on the RT 4.09 sample and the commercially obtained 5-HMF (Sigma Aldrich, Cat No. W501808). We found that the HNMR for our RT 4.09 sample (Fig. 9A) had similar chemical shifts to the predicted HNMR chemical shifts for 5-HMF (Fig. 7A). The RT 4.09 HNMR had peaks with chemical shifts of 4.63, 6.60, 7.40 and 9.5 ppm (Fig. 9A), while the predicted 5-HMF HNMR (Fig. 7A) had peaks with chemical shifts of 3.3, 4.6, 6.6, 7.4 and 9.5. With most of the peaks being identical between the predicted 5-HMF and the RT4.2 sample these peaks must correspond to the protons at locations 7,4,3 and 6 in the 5-HMF molecule (Fig. 8). The RT 4.09 NMR analysis showed that the predicted peak at 3.3, corresponding to the hydroxyl group at position 8 (Fig. 8), was absent. This is likely due to the use of deuterated methanol as the solvent, which facilitates proton exchange with the hydroxyl group, rendering it undetectable by the NMR machine. To follow up the HNMR we also performed a CNMR on the RT 4.09 sample (Fig. 9B) and similarly we found chemical shifts that matched the predicted shifts almost perfectly, with the shifts of 56,109, 123,152,161,178 in the CNMR almost perfectly matching the predicted shifts at locations 7,4,3,2,5,6 in the 5-HMF molecule (Fig. 8). With the predicted chemical shifts for 5-HMF matching the chemical shifts for the RT 4.09 sample so closely we also performed a HNMR and CNMR on the purchased 5-HMF and found the chemical shifts where identical to our RT 4.09 sample (Fig. 9C and D). Two chemical shifts were also identified in the RT4.2 and 5-HMF HNMR and one in the CNMR that were not present in the predicted HNMR and CNMR. In the HNMR the chemical shift of 4.97 was identified as D2O and the 3.33 peak which was used as the reference was the chemical shift for deuterated methanol. Deuterated methanol was also found in the CNMR for both with a chemical shift of 47.75 and used as the reference. To further reinforce that the RT 4.09 sample was indeed 5-HMF, we decided to spike the 5-HMF sample with an equal volume of the RT 4.09 sample and determine if there were any new chemical shifts not identified previously. We found that the HNMR and CNMR chemical shifts in the 5-HMF spiked sample were identical (Fig. 10A and B) to the chemical shifts for both the RT4.2 sample and the purchased 5-HMF sample indicating that the RT 4.09 sample is 5-HMF.

### 3.5. 5-HMF stimulates SOD2 expression

**Fig. 11.**
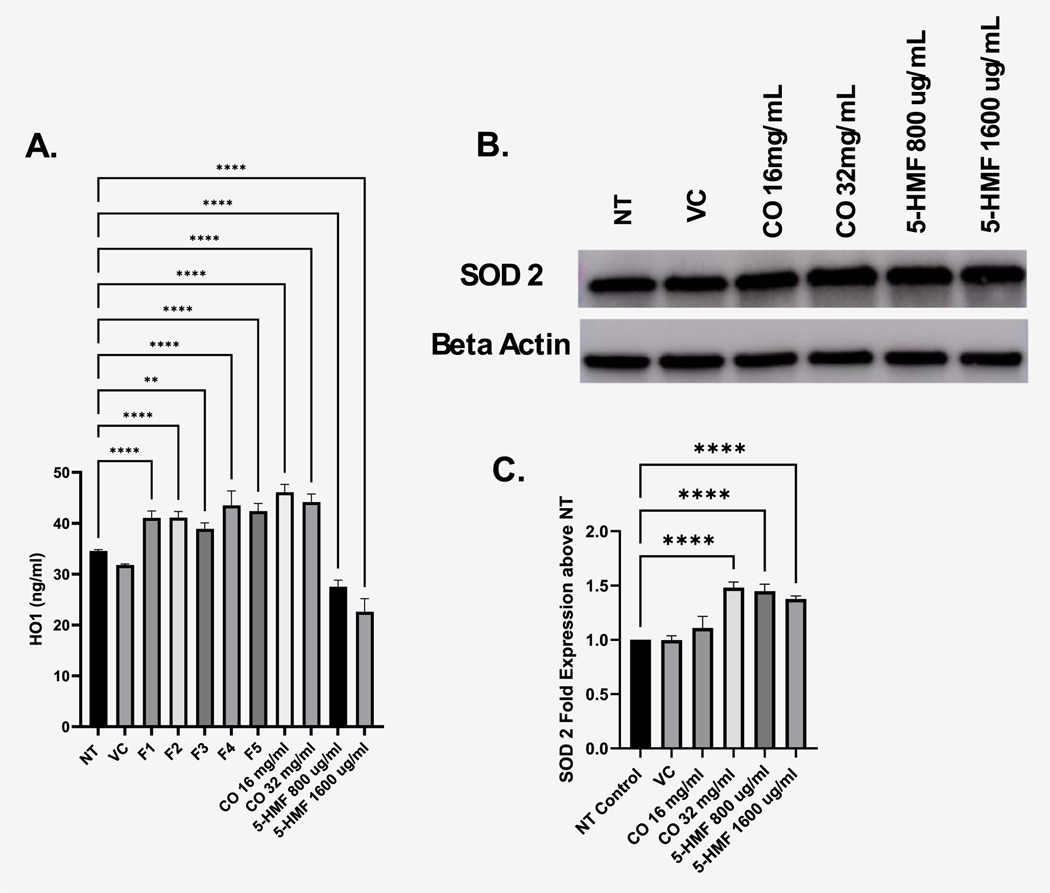
HO1 ELISA and SOD2 Immunoblot after treatments with *C. officinalis* (CO) and 5-HMF **(A)** Whole cell lysate was collected after corresponding treatments and HO1 concentration was determined by commercial ELISA. Average HO1 levels are shown as mean ± SEM. P < 0.05 as determined by one way ANOVA test (N = 3) Post hoc analysis performed by Dunnett’s multiple comparisons test. **(B)** Western analysis of SOD2. Western analysis was performed on protein lysates following various treatment condition, Treatment conditions are shown above lanes. **(C)** Densitometric analysis of immunoblots following 3 independent experiments as determined by ImageJ. Average SOD2 levels are shown as mean ± SEM. P < 0.05 as determined by one way ANOVA test (N = 3) Post hoc analysis performed by Dunnett’s multiple comparisons test.

We previously provided evidence to suggest that the activation of the Keap1/Nrf2 pathway is *C. officinalis*’s mechanism of action within the pancreatic β-cell 1.1B4 cell line (Sharp-Tawfik et al., 2022). Therefore, determining whether 5-HMF can activate this pathway is crucial to understanding its role in *C. officinalis*’s biological effects. We tested this by examining the expression of two downstream targets of the Keap1/Nrf2 pathway: HO1 and SOD2 (Mukherjee et al., 2025). HO1 expression was examined by ELISA to determine if 5-HMF increases HO1 expression as well as determine if there were any differences in expression between the un-purified fractions from the column chromatography (F1-F5) and purified 5-HMF. Interestingly, we found that 5-HMF caused a significant decrease in expression of HO1 (Fig. 11A) while *C. officinalis* extract and the unpurified fractions all significantly increased expression. Furthermore, fractions F4 and F5 induced the highest expression of HO1 which was unexpected, as these fractions exhibited the smallest peaks and consequently, the lowest concentrations of the RT 4.09 peak, which we identified as 5-HMF.RT 4.09. This indicates that 5-HMF is not responsible for *C. officinalis*’s induction of HO1 expression. We also found that exogenous application of 5-HMF to 1.1B4 cells did not increase cell viability as determined by MTT assay (data not shown).

As 5-HMF had been shown to activate the Keap1/Nrf2 pathway in the mouse brain in vivo (Ya et al., 2017), in addition to our prior results demonstrating that *C. officinalis* extract can also promote this pathway in a pancreatic β-cell line, we measured the effect of 5-HMF on the 1.1B4 cell line and measured downstream targets of the Keap1/Nrf2 pathway that were identified by our prior results. 5-HMF significantly increased SOD2 expression in a dose dependent manner similar to the significant increase shown by whole *C. officinalis*. (Fig. 11B and C). This indicates that although 5-HMF can induce a significant increase in expression of SOD2, it may only be partially responsible for *C. officinalis*’s biological effects in pancreatic β-cells.

### 3.6. Quantitative proteomic analysis of 5-HMF treated NOD murine islets

To further validate the biological impact of 5-HMF in primary cells, we conducted a proteomic analysis of 5-HMF treated isolated murine islets. Murine islets were isolated from 8 wk old female NOD mice and cultured 24 h prior to treatment with 5-HMF at both a high and low dosage. Following an 18 h exposure, protein was isolated from the murine islets and then processed for mass spectrometry as detailed in materials and methods. Significant and differentially expressed proteins were identified between treatment groups. Across all comparison treatment groups (No treatment vs. High Dose, No treatment vs. Low Dose, and High Dose vs. Low Dose) there were approximately 65,000 peptides and 6500 proteins identified by our analytical methods following the applied peptide filter and utilized for the statistical analysis. For the scope of this manuscript, we focused the reporting on those proteins associated with the Keap1/Nrf2 pathway. The results from this proteomic analysis revealed that there was a significant increase in the abundance of Nrf2 as well as several downstream targets such as SOD2, GCLC (Morgenstern et al., 2024), GSTA3 (Xiao et al., 2019), and GSTA4 (Ma et al., 2025) (Fig. 13). Nrf2 abundance was increased in a dose-dependent manner at both the high (log2 fold change of 1.48) and low (log2 fold change of 0.81) dose 5-HMF treatment groups. Concordantly, Keap1 expression was significantly decreased (log2 fold change of − 1.2) at the high 5-HMF dose as well. Increased abundance of SOD2, GSTA 4, GSTA 3, and GCLC was found significantly increased at the high dose only. Interestingly, as observed in a manner similar to that found in the 1.1B4 cell line (Fig. 11A), HO-1 was decreased in a dose dependent manner at both the high (Log2 fold change of − 1.8) and low (Log2 fold change of − 0.89) dose 5-HMF treatment groups. The increased abundance of Nrf2 matched with decreased Keap1 along with increased downstream antioxidant targets suggests that 5-HMF is promoting stimulation of the Keap1/Nrf2 pathway in primary islets.

### 3.7. 5-HMF treatment decreases insulin secretion in the NIT-1 cell line

Our proteomic analysis also revealed decreased intracellular insulin (INS1) expression in primary islets treated with 5-HMF compared to the NT group (Fig. 13). To confirm these findings for insulin secretion, we decided to use a transformed β cell line known as NIT-1, since the 1.1B4 cell do not have measurable insulin secretion. The NIT-1 cell line is derived from the NOD murine pancreatic β-cells and secrete insulin in response to glucose (Hamaguchi et al., 1991). As this model closely mimics the primary islets and is suitable for a single target ELISA investigating insulin secretion, we treated the NIT-1 cells with a high (1600 μg/ml) and low (800 μg/ml) dose of 5-HMF under 7.0 mM glucose conditions. 5-HMF treatment resulted in a significant decrease in insulin secretion at both dosages as compared with untreated NIT-1 cells (Fig. 14), which is consistent with the decreased abundance of intracellular insulin from 5-HMF treated murine pancreatic islets as measured from the quantitative proteomics (Fig. 13, INS1).

## 4. Discussion

Our prior published findings revealed that *C. officinalis* treatment activated the Keap1/Nrf2 pathway and downstream targets such as HO1 and SOD2 in the 1.1B4 pancreatic β-cell line (Sharp-Tawfik et al., 2022). We then provided evidence demonstrating that *C. officinalis* extract delivered by oral gavage could delay the onset of T1D and inhibit pancreatic insulitis in the NOD mouse model (Fletcher et al., 2024). One important question remaining was which compound or combination of compounds was responsible for *C. officinalis’s* biological effects observed in vivo and in vitro. We hypothesized that either loganin or morroniside was responsible, as both compounds have been isolated from *C. officinalis* and are abundantly present in its extract, as shown in our study (Sharp-Tawfik and Burkhardt, 2019). Additionally, both have been recognized for their biological significance (Zhang et al., 2017) (Cheng et al., 2020) but interestingly our results demonstrated no stimulatory increase in the viability of the 1.1B4 cell line. In addition, neither promoted increased HO-1 expression (Fig. 1) which is an important Nrf2 target. We then used an analytical HPLC system to reveal that although both loganin and morroniside were abundant in whole *C. officinalis* extract, there were many other high intensity peaks indicating additional prominent compounds (Fig. 2). To initially screen individual compounds for biological activity, fractionation of the whole *C. officinalis* extract was performed using a column chromatography method followed by purification by HPLC (Figs. 3–5) and then subsequent biological testing of individual fractions. Interestingly we found the fractions had a dose dependent effect on viability proportional to the intensity of the RT 4.09 peak (Fig. 4). We then purified the RT 4.09 peak by HPLC and utilized NMR to identify the compound as 5-HMF (Figs. 7–10).

5-HMF has been extensively investigated and demonstrated to be a biologically active compound isolated from *C. officinalis* in other cell types but not in pancreatic β-cells (Cao et al., 2013; Jiang et al., 2015). Our report would be the first to our knowledge, reporting the effect of 5-HMF on a pancreatic β-cell line or murine islets. Interestingly, our prior HPLC/MS analysis of *C. officinalis* did not identify 5-HMF as a highly abundant compound. In fact, upon examination of *C. officinalis* from 4 independent commercial sources, the primary abundant compounds were identified as Morroniside, Morroniside diglycoside, Loganin, Cornuside II, Malic Acid, Ellagic Acid, Loganic Acid, Gallic Acid, Swereoside, and Tartaric Acid (Sharp-Tawfik and Burkhardt, 2019) which stands in contrast to our most recent findings demonstrating that 5-HMF was the most abundant compound by far. 5-HMF has also been found to be biologically active in vivo, demonstrating its ability to prevent induced liver injury by suppressing oxidative stress (Han et al., 2017). Furthermore, 5-HMF may be clinically relevant to diabetes as 5-HMF can prevent high glucose-induced oxidative stress in human umbilical vein endothelial cells in vitro (Cao et al., 2013). Our previous findings demonstrated that *C. officinalis* strongly activates the antioxidant response by stimulating the Keap1/Nrf2 pathway in the 1.1B4 cell line mirroring 5-HMF’s biological activity in the liver (Sharp-Tawfik et al., 2022). For this reason, we determined if 5-HMF had the same biological effect as whole *C. officinalis* extract and found that 5-HMF was only partially responsible for *C. officinalis*’s biological effect in the 1.1B4 cell line as it significantly upregulated SOD2 but not HO1 expression (Fig. 11. These results were further validated by proteomic analysis of primary islets, which when treated with 5-HMF showed increased levels of SOD2, Nrf2, GCLC, GSTA3, and GSTA4; but expectedly decreased levels Keap1 but also the paradoxical result of decreased HO-1 (Fig. 13). Interestingly, this suggests that while 5-HMF is biologically active in the 1.1B4 cell line and pancreatic murine islets, it does not solely account for *C. officinalis*’s biological effect providing evidence that *C. officinalis* may have a combinatorial effect.

**Fig. 12.**
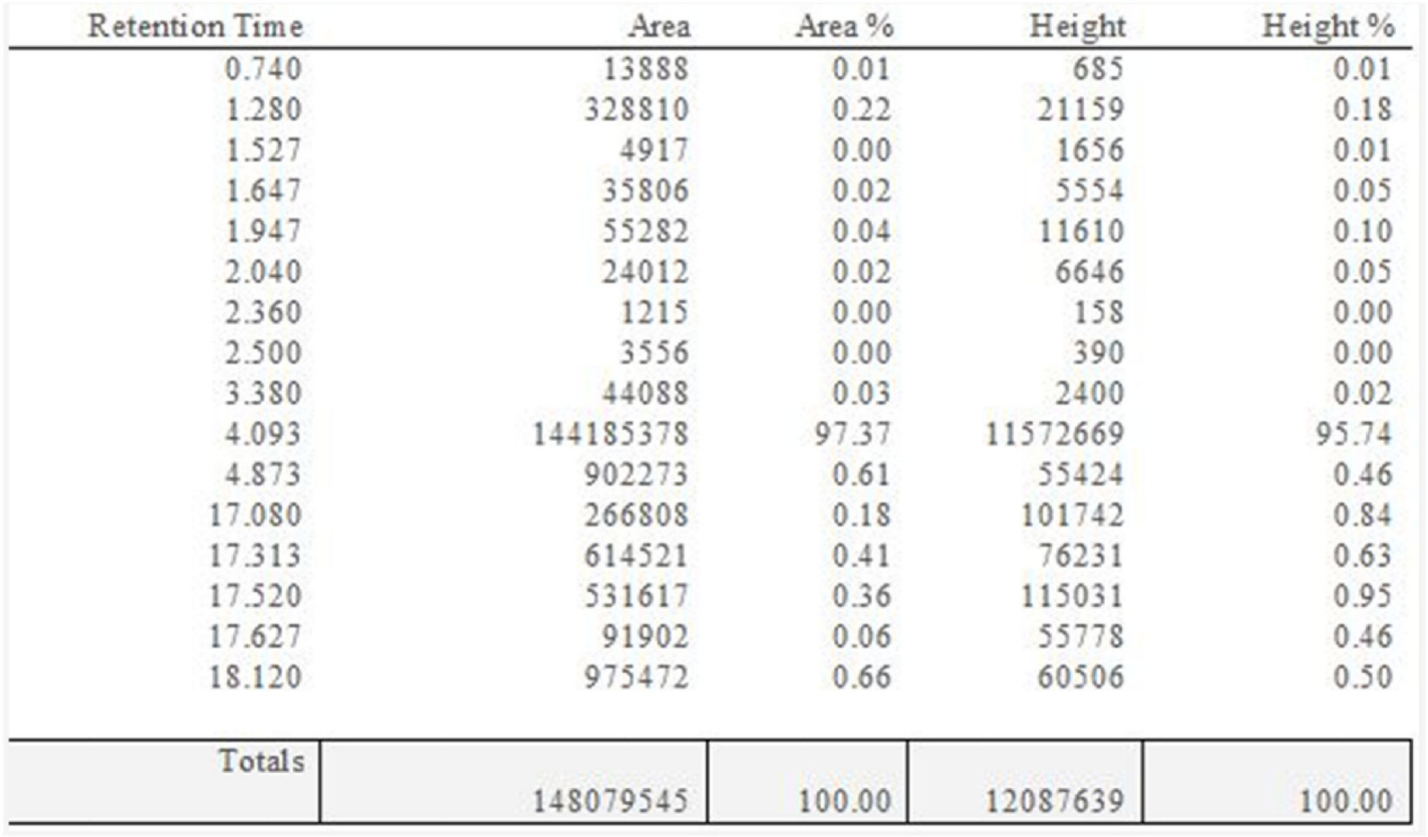
Percent area of purified RT 4.09 fraction relative to total area of all peaks Table showing the Area, Area %, Height and Height Percent of all peaks in the purified RT 4.09 sample.

A conflicting result in our investigation was the lack of increased cell viability as observed by MTT assay when 1.1B4 cells were stimulated with commercially received 5-HMF alone in contrast to the increased viability measured when applied with purified fraction from retention time 4.09 (Fig. 4B). We confirmed that our isolated fraction contains 5-HMF at 97.37 % purity based on area % (Fig. 12); in addition, our HNMR comparison shows identical spectra for both the commercial and lab-purified 5HMF samples, indicating that there are no structural differences between the 5-HMF we obtained and the commercially available 5-HMF (Figs. 7–10). Although our isolated 5-HMF sample has a high degree of purity, there are still trace amounts of additional compounds which may account for differences in the MTT assay results between the commercially received 5-HMF and our 5-HMF samples purified from whole *C. officinalis* extract. While we have shown that 5-HMF activates the Keap1/Nrf2 pathway its effect on β-cell viability as measured by the MTT assay may only be partial. The full enhancement in viability observed during the MTT assay after treatment with *C.officinalis* likely requires the combined action of 5-HMF and other trace elements present in the extract. This supports our conclusion that 5-HMF, while biologically relevant to activation of Keap1/Nrf2, is one of multiple compounds that contribute to *C. officinalis* mediated pancreatic β cell viability.

**Fig. 13.**
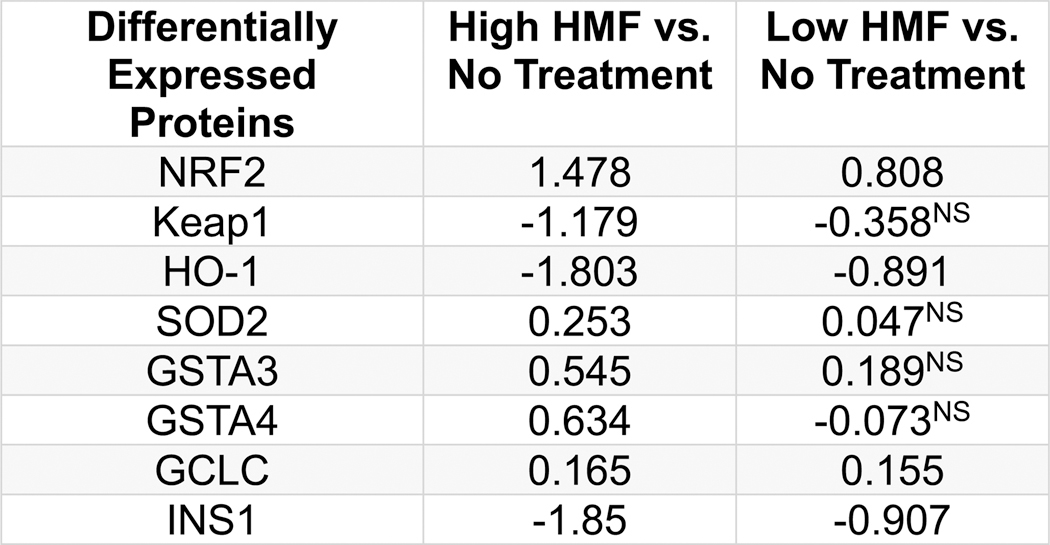
Differentially expressed proteins in 5-HMF treated NOD murine islets. Table showing log2 fold change differences between 5-HMF treated islets vs No Treatment islets (NT) as determined by proteomic analysis. High and Low 5-HMF indicates murine NOD islets treated with 1600 and 800 μg/ml, respectively. Log2 fold differences are all significant unless designated by NS.

**Fig. 14.**
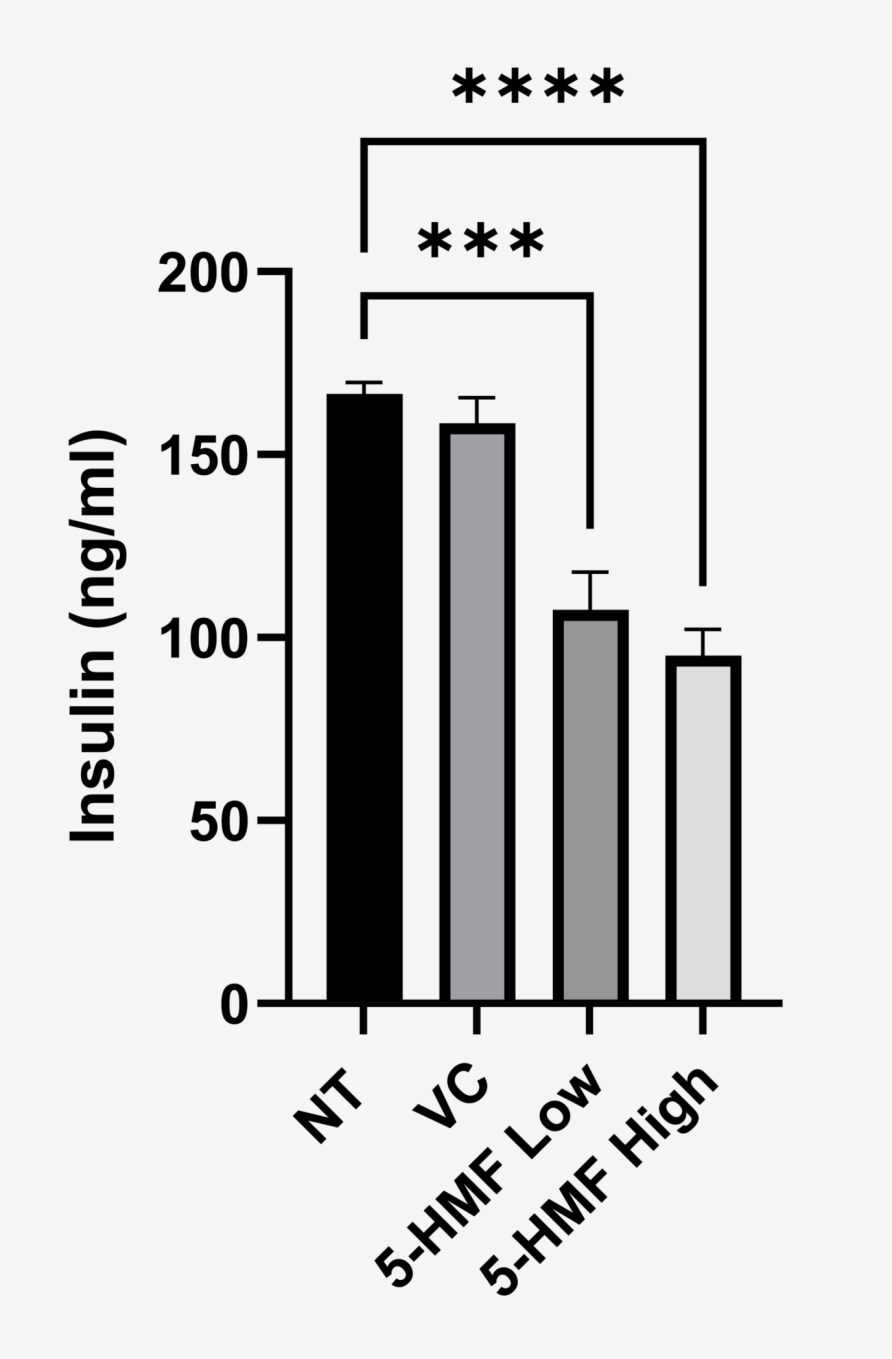
Insulin secretion from HMF treated NIT-1 cells. High and Low 5-HMF indicate NIT-1 cells treated with 1600 and 800 μg/ml of 5-HMF. Insulin concentration was determined by commercial ELISA. Average insulin levels are shown as mean ± SEM. P < 0.05 as determined by one way ANOVA test (N = 4) Post hoc analysis performed by Dunnett’s multiple comparisons test. NT and VC denotes No Treatment and Vehicle Control, respectively.

Another intriguing finding from our results is the selective 5HMF-stimulated induction of Nrf2 regulated antioxidant expression. As mentioned earlier, our prior publication strongly indicated that *C. officinalis* extract stimulated the Keap1/Nrf2 pathway with induction of downstream effectors such as HO1 and SOD2. While our current results did indicate that the unpurified extracts of *C. officinalis* could stimulate HO1 (Fig. 11B), isolated 5-HMF decreased HO1 in the 1.1B4 cell line. However, isolated 5-HMF increased SOD2 in both 1.1B4 cells and primary islets, as well as additional Keap1/Nrf2-associated proteins including GCLC, GSTA3, GSTA4, and Nrf2 in primary islets (Fig. 13); increased abundance of these downstream targets indicates an activation of the Keap1/Nrf2 pathway. The observed discrepancy may stem from the activation of additional pathways induced by 5-HMF. Our proteomic analysis of primary islets stimulated with 5-HMF revealed a marked upregulation of the Keap1/Nrf2 pathway, alongside several other signaling pathways. Notably, existing literature highlights that various post-translational modifications of Nrf2, as well as epigenetic alterations within the genome, can significantly influence Nrf2 signaling and modulate the expression of its downstream targets (Yang et al., 2025). Furthermore, existing literature suggests that knockdown of the key chromatin remodeling enzyme BRG1 enhances Nrf2 binding to the HO1 promoter, but not to the NQO1 promoter (Song et al., 2020). Although both genes are downstream targets of the Keap1/Nrf2 pathway, this differential effect implies that chromatin remodeling may selectively regulate specific Nrf2-responsive elements. High concentrations of 5-HMF may potentially activate pathways that cause epigenetic changes, preventing Nrf2 from being able to promote HO1 expression while still being able to upregulate the other Nrf2 targets, potentially explaining our observed results of a decrease in expression of HO1 while having an increase of expression of other Nrf2 targets.

An additional compelling observation was that treatment with 5-HMF resulted in a significant decrease in insulin secretion from pancreatic β cells. While activation of the antioxidant response in β cells is generally considered a strategy to enhance cellular viability, the observed reduction in insulin secretion presents a potentially confounding outcome. Notably, reactive oxygen species (ROS) signaling plays a critical role in glucose-stimulated insulin secretion (GSIS), and the upregulation of antioxidant pathways can paradoxically impair this process. Interestingly, The literature demonstrates that increased expression of antioxidant enzymes reduces intracellular ROS levels, thereby diminishing the ROS available to induce GSIS (Pi et al., 2010). Given our findings that 5-HMF robustly activates the Keap1/Nrf2 pathway, this strong induction of the antioxidant response may lead to an overall reduction in ROS within the cell, offering a plausible explanation for the observed decrease in insulin production.

5-HMF induced Nrf2 activation may have potential therapeutic value for enhancing pancreatic β-cell survival particularly when exposed to the hyperinflammatory environment of insulitis accompanying T1D onset. SOD2 is a critical antioxidant enzyme that scavenges oxygen radicals through oxidation and reduction cycles resulting in decreased oxidative stress (Zheng et al., 2023). Since oxidative stress plays a crucial role in pancreatic β-cell dysfunction and directly contributes to the development of clinical diabetes (Dinic et al., 2022), upregulating antioxidant enzymes like SOD2 in the β-cell may provide a novel method of diabetes treatment. Furthermore, detrimental polymorphisms in the human SOD2 gene inhibiting processing of this antioxidant enzyme have been shown to be associated in T1D patients exhibiting various complications such as diabetic nephropathy (Houldsworth et al., 2015). Approaches enhancing targeted antioxidant expression in the endocrine pancreas may diminish the harmful milieu associated with T1D.

Future studies will need to be performed to demonstrate the beneficial role of 5-HMF in type 1 diabetes. To address the limitations found in this investigation and address which compounds from *C. officinalis* are needed in conjunction with 5-HMF to achieve *C. officinalis*’s full biological effects. For example, one critical experiment is to determine if 5-HMF can upregulate SOD2 expression in the endocrine pancreas of the NOD mice and delay diabetic onset.

## Data Availability

Data will be made available on request.
